# Abscess formation mimicking disease progression, in a patient with metastatic renal cell carcinoma during sunitinib treatment

**DOI:** 10.1186/1477-7819-8-45

**Published:** 2010-05-28

**Authors:** Vasiliki Michalaki, Nikolaos Arkadopoulos, Agathi Kondi-Pafiti, Constantine Gennatas

**Affiliations:** 1 Oncology Clinic, Second Department of Surgery, University of Athens, Aretaieion Hospital, 78, V. Sofias av, 115 28, Athens, Greece; 2 Second Department of Surgery, University of Athens, Aretaieion Hospital, 78, V. Sofias av, 115 28, Athens, Greece; 3 Histopathology Department University of Athens, Aretaieion Hospital, 78, V. Sofias av, 115 28, Athens, Greece

## Abstract

**Background:**

Renal cell carcinoma (RCC) represents approximately 3% of all adult cancers and is more common in males. Systemic treatment for RCC has improved following the introduction of tyrosine kinase inhibitors, such as sunitinib. The molecular targets of sunitinib are receptor tyrosine kinases (RTKs). Moreover, sunitinib has an additional anti-angiogenic effect through its inhibition of the vascular endothelial growth factor receptor activation.

**Case presentation:**

We present a case of intra-abdominal abscess formation mimicking disease progression, in a patient with metastatic renal cell carcinoma during sunitinib treatment.

**Conclusion:**

In the advancing era of molecular therapy of solid tumours, sunitinib has demonstrated significant efficacy in the post-cytokine setting treatment of metastatic renal cancer. Concurrently, however, increasing evidence has emerged to indicate that this class of drugs exert profound immunomodulatory effects on T cells and play major roles in immune tumor surveillance.

## Background

The treatment of advanced RCC is undergoing a paradigm shift with the recent introduction of anti-angiogenic therapy that either directly inhibits vascular endothelial growth factor or disrupts signal transduction favorable to vascular development through multi-kinase inhibitors. Angiogenic inhibitors have been found to increase survival and are approved in advanced renal cell carcinoma [1,2]. Consequently, most of these patients will routinely receive tyrosine kinase inhibitors, such as sunitinib.

Sunitinib is an orally administered, small molecule inhibitor of multiple receptor tyrosine kinases implicated in tumour growth, angiogenesis, and metastatic progression. In addition, the targets of sunitinib involve vascular endothelial growth factor receptors (VEGFR1, VEGFR2 and VEGFR3), platelet-derived growth factor receptors (PDGFRα and PDGFRβ) and the like. We describe a case of intra-abdominal abscess formation mimicking disease progression during sunitinib treatment.

## Case presentation

A 62-year-old patient diagnosed with a high-grade clear-cell renal carcinoma in 1991 and was treated by left nephrectomy and surrenalectomy. Fourteen years later, relapsed on the lungs and had been administered interferon alfa. The patient was regularly followed up and had regular scans that did show stabilization of the disease in the lungs for two years. In December 2007 chest computerized tomography (CT) disclosed the progression of lung metastases. Sunitinib was initiated in January 2008 as a standard regimen (50 mg/day for 4 weeks every 6 weeks) for pulmonary metastases. Patient had a radiographic response and prolonged progression free survival of fourteen months; side effects were manageable and included grade 2 hypertension. After five cycles, the patient was admitted to the hospital due to complaints of fatigue and left sided flank pain. The systolic and diastolic blood pressures were 110 mmHg and 60 mmHg, respectively, pulse rate was 90 per min and respiratory rate was 20 per min. The body temperature was 37.2°C.

Laboratory studies were conducted immediately after the patient's arrival at the hospital. He had anemia (Hb 98 g/L) (normal range: 140-180) and thrombocytopenia (133 × 10^9^/L) (normal range: 150-450), but a WBC count was normal (6.15 × 103/mm^3^) with 74% neutrophils. Other laboratory findings were presented as elevated serum levels of CRP (21 mg/L) (normal range:< 5), ALP (416 IU/L) (normal range: 96-250), and slightly increased creatinine (1.43 μmol/L) (normal range: 0.5-1.2).

**Figure 1:**
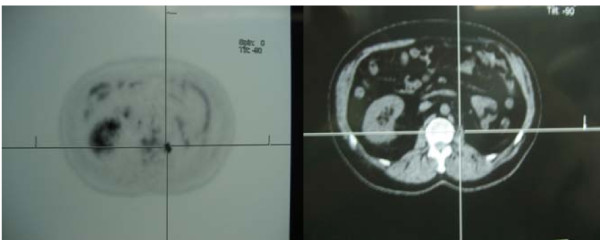
**PET - CT demonstrating an area of increased uptake in the left paravertebral area**.

**Figure 2:**
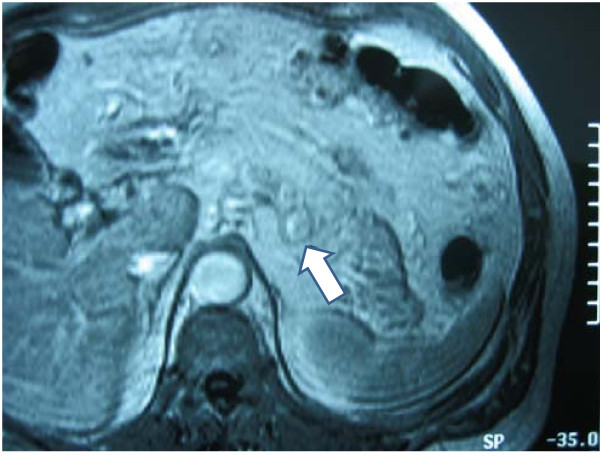
**MRI scan (T2 image) demonstrating the lesion that corresponds to the area of increased PET uptake**.

Fluorodeoxyglucose positron emission tomography (FDG-PET-CT) scans demonstrated an area of increased uptake in the left paravertebral area (Figure 1). MRI scan (T2 image) demonstrated the lesion that corresponded to the area of increased PET uptake (Figure 2).

The patient underwent a diagnostic laparoscopy in May 2009. Intra-operative biopsy of the lesion was performed; the pathology was consistent with an abcess without evidence of malignancy. After an uneventful postoperative course, the patient was discharged on the 10th day after surgery and chemotherapy with sunitinib was restarted. Three months postoperatively there was no evidence of recurrent disease.

## Discussion

Renal cell cancer (RCC) is a relatively uncommon malignancy. When the disease is localized is curable by surgery; however, locally advanced or metastatic disease is not curable in most cases and until recently had a limited response to drug treatment. Historically, biologic response modifiers or immunomodulating agents were tested in clinical trials based on observations that some cases of RCC can spontaneously regress. Responses have been observed with interferon alfa, but with little effect on overall survival.

The use of targeted therapies has substantially improved outcomes for patients with advanced renal cell carcinoma [3,4].

Sunitinib malate is an oral multi-kinase inhibitor targeting several receptor tyrosine kinases (PDGFRalpha and PDGFRbeta; VEGFR1, VEGFR2 and VEGFR3; KIT, FLT3, CSF-1R and RET) that was approved by the FDA in 2006 for treatment of metastatic renal cell carcinoma. In a randomized phase III trial, sunitinib prolonged median progression-free survival (11 months) in comparison to interferon-alpha (5 months); corresponding to a hazard ratio of 0.42 (95% confidence interval: 0.32 to 0.54; P < 0.001) for patients with advanced renal cell cancer. Sunitinib was also associated with a higher objective response rate than interferon-alpha (31% vs. 6%; P < 0.001) [2].

The most common toxicities with sunitinib are hand-foot syndrome, rash, fatigue, hypertension, and diarrhea.

Concurrently, however, increasing evidence has emerged to indicate that TKIs such as sunitinib, exert profound immunomodulatory effects on T cells and antigen-presenting cells, such as dendritic cells, which play major roles in immune tumor surveillance. Targeted tyrosine kinase inhibitor therapy may thus control cancer cell growth both directly and indirectly by changing the immunologic microenvironment. These side-effects of therapy on normal vasculature type may lead to rare complications such as the abscess formation in the present case. However, physicians should have in mind that occasionally disease extension to unexpected anatomical sites does occur, causing unusual clinical pictures. For the differential diagnosis between these conditions, CT scan is considered to be the imaging study with the highest accuracy and efficiency [5,6]. Not only can it be of great help in diagnosis, but also in evaluating the extension of involvement. Furthermore, an approach for drainage of abscesses can be made on CT results. However, sometimes an exploratory laparotomy is necessary to reveal the cause. Nonetheless, in our case, CT findings were not sufficient for the diagnosis and the cause of imaging findings was unclear until laparotomy.

In summary, a case of an intra-abdominal abscess formation mimicking disease progression during sunitinib treatment, was presented. After an uneventful postoperative course, the patient was discharged on the 10th day after surgery and chemotherapy with sunitinib was restarted.

Although stage IV disease is generally not considered curable, the literature and clinical experience identifies many long-term survivors, reflecting the unpredictable nature of this malignancy. Research is directed toward defining the optimal use of these new agents.

## Consent

Written informed consent was obtained from the patient for publication of this case report and accompanying images. A copy of the written consent is available for review by the Editor-in-Chief of this journal.

## Competing interests

The authors declare that they have no competing interests.

## Authors' contributions

Conception and design: VM, and CGG. Provision of study material: CGG, NA, AKP. Collection and assembly of data: VM, NA. Manuscript writing: VM
